# Conducting proportional meta-analysis in different types of systematic reviews: a guide for synthesisers of evidence

**DOI:** 10.1186/s12874-021-01381-z

**Published:** 2021-09-20

**Authors:** Timothy Hugh Barker, Celina Borges Migliavaca, Cinara Stein, Verônica Colpani, Maicon Falavigna, Edoardo Aromataris, Zachary Munn

**Affiliations:** 1 JBI, The University of Adelaide, 55 King William Rd, North Adelaide, SA 5006 Australia; 2 Programa de Pós-Graduação em Epidemiologia, Universidade Federal do Rio Grande do Sul, Porto Alegre, Rio Grande do Sul Brazil; 3 Hospital Moinhos de Vento, Porto Alegre, Rio Grande do Sul Brazil; 4 Department of Health Research Methods, Evidence and Impact, McMaster University, Hamilton, ON Canada

**Keywords:** Research synthesis, meta-analysis, Proportional meta-analysis, Systematic reviews

## Abstract

**Background:**

Single group data present unique challenges for synthesises of evidence. Proportional meta-analysis is becoming an increasingly common technique employed for the synthesis of single group data. Proportional meta-analysis shares many similarities with the conduct and reporting of comparative, or pairwise, meta-analysis. While robust and comprehensive methods exist detailing how researchers can conduct a meta-analysis that compares two (or more) groups against a common intervention, there is a scarcity of methodological guidance available to assist synthesisers of evidence in the conduct, interpretation, and importance of proportional meta-analysis in systematic reviews.

**Main body:**

This paper presents an overview targeted to synthesisers of evidence and systematic review authors that details the methods, importance, and interpretation of a proportional meta-analysis. We provide worked examples of how proportional meta-analyses have been conducted in research syntheses previously and consider the methods, statistical considerations, and presentation of this technique.

**Conclusion:**

This overview is designed to serve as practical guidance for synthesisers of evidence in the conduct of proportional meta-analyses.

## Background

Meta-analysis is a common method of synthesis of quantitative data from two or more independent yet comparable studies included in a systematic review [1–3]. The majority of meta-analyses reported in the medical literature are comparative pairwise (two group) analyses and are largely used to establish the effect of an intervention (or exposure) compared with a comparator to generate a pooled estimate of effect, expressed as a risk ratio, odds ratio, incidence rate ratio or weighted mean difference (among many others) [4]. There are also approaches for multiple group meta-analysis (i.e network meta-analysis) and for single group meta-analysis (proportional meta-analysis, meta-analysis of means, correlation coefficients, incidence rates etc).

As with pairwise meta-analysis (where data for two unique groups are synthesised to produce a pooled estimate of effect), the goal of the meta-analysis of proportions is the generation of a single summary estimate and its variance. Termed ‘proportional meta-analysis’, this method of data synthesis allows for calculation of a pooled, overall proportion from a number of individual proportions. Any dichotomous data or data that can be reported as a percentage can be included in a proportional meta-analysis. These meta-analyses are most commonly used to quantify disease occurrence in populations to answer questions related to both the prevalence and incidence of disease and are typically presented in systematic reviews dealing with prevalence and cumulative incidence data. However, proportional meta-analysis may also be used for review types other than prevalence, including when trying to identify evidence regarding the effectiveness of treatments or interventions, overall prognosis or baseline risk [5, 6]. The purpose of this paper is to introduce the concepts of proportional meta-analysis, practical guidance for systematic reviewers including a proportional meta-analysis in their review, the statistical considerations specific to this analysis and how this format of synthesis can be utilised by systematic reviewers across different review types with different questions and indications.

## Main body

### Systematic reviews of prevalence and cumulative incidence

In terms of systematic reviews, the most common usage for a proportional meta-analysis is to assist in answering a question related to the prevalence or the incidence of disease in a population. There has been a 10-fold increase in the number of systematic reviews of prevalence and incidence published from 2007 to 2017 with the terms “systematic review” and “prevalence” in the title, with 64.7% of these reviews performing a proportional meta-analysis to synthesise prevalence estimates [7]. There are several measures of disease frequency: point-prevalence is an indicator of who has a disease at a certain point in time; period-prevalence indicates who has the disease within a given time frame; and cumulative incidence is an indicator of how often the disease develops (i.e. new cases of a disease among a population) over a certain period [8, 9]. These metrics are reported in the literature in terms of their proportions, that is the number of cases divided by the total population number [4]. Lifetime prevalence (a commonly reported period-prevalence), is the proportion of a population who has ever had the disease of interest. Given the nature of the estimates and that they are all have temporal dependence, for any condition, lifetime prevalence is expected to be higher than either point or a more restricted period prevalence. Prevalence and cumulative incidence estimates are normally generated through observational studies and proportions are the typical format in which data is likely to take when conducting a systematic review of prevalence or cumulative incidence [8, 9]. Prevalence and incidence reviews may not only address the prevalence or incidence of disease itself, but may also answer questions related to many other types of variables. For instance, systematic reviews of prevalence with associated proportional meta-analyses have been conducted when investigating the symptoms of a disease [10]; pre-existing conditions [11], events [12] and cultural practices or behaviours [13]. Proportional-meta analyses can also provide quantitative descriptions regarding specific covariates of disease frequency, such as the geographical distribution of a variable [14] or the difference in frequency of a particular disease between males and females [15].

Proportional meta-analysis in these review types provides a single summary estimate of the prevalence or incidence of a condition across the included studies. The appropriateness of conducting meta-analysis of this type of data is contentious, as the individual studies contributing to such a meta-analysis commonly have been conducted in different contexts and the prevalence and cumulative incidence estimates produced from these studies are reflective of unique population characteristics [16].

This raises some concerns when proportional meta-analysis assumes homogeneity, and an average estimate across many different populations may be of little clinical use [4]. However, for a systematic review with the goal of estimating global disease burden, a proportional meta-analysis is argued to still be an appropriate and effective method of data synthesis [17]. For example, if there is interest in investigating the likely disease burden for a certain population and if no primary studies have been conducted in that population of interest, then it may be prudent to find studies that are similar to the population of interest that report proportional data, and synthesise the data with proportional meta-analysis [18].

#### Review Example 1

**Fig. 1:**
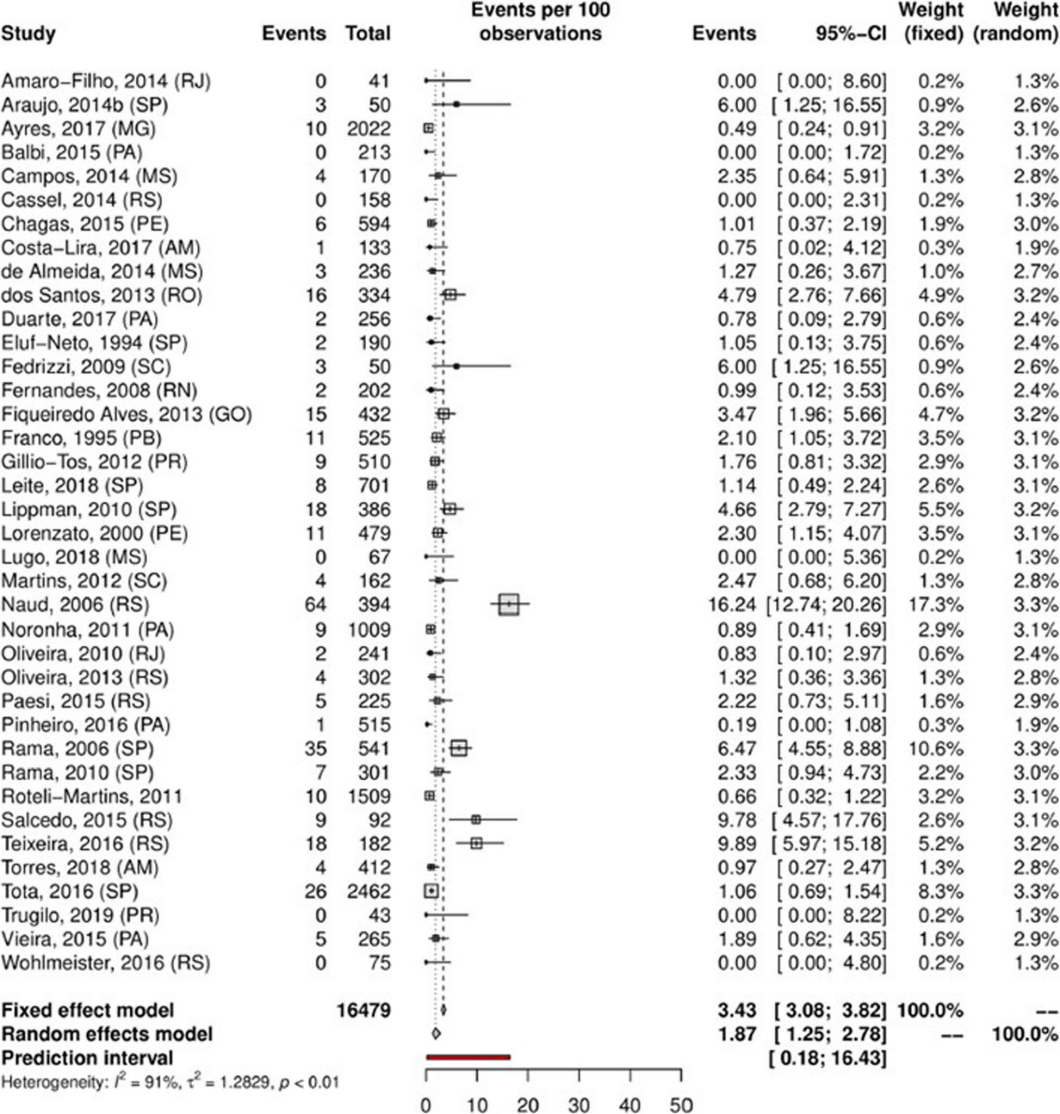
Example prevalence proportional meta-analysis investigating overall prevalence of cervical infection by HPV-18 in Brazil. Created in R, package meta. Reproduced under Creative Commons from: Colpani, V et al. “Prevalence of human papillomavirus (HPV) in Brazil: A systematic review and meta-analysis”. PloS one 15.2 (2020): e0229154 [20]

Understanding and estimating the prevalence of a condition or practice can also be beneficial during the planning and development of new health resources on a societal scale. When governments are required to allocate resources to health expenditure, they must undertake significant investigation or “strategic purchasing” of any new decision made [19]. One of the first considerations for any new decision made is the assessment of population needs [19]. By providing a pooled proportional estimate for a prevalence variable over a designated time period, the results of a proportional meta-analysis (as a component of a well conducted and rigorous systematic review of prevalence and incidence) [9], can enable governments, policy makers and health professionals to make informed decisions regarding the development and delivery of health services [8, 9, 16]. For example, a systematic review was conducted to assess the prevalence of human papillomavirus (HPV) in Brazil to assist health service policy and planning [20]. The authors identified 139 eligible studies comprising more than 50,000 people and were able to conduct a number of proportional meta-analyses by region, population and anatomical site. Figure 1 below is an example forest plot and proportional meta-analysis reporting the prevalence of cervical infection by HPV-18 as 1.87% (Random effects model, 95% CI 1.25–2.78). Based on this research, the authors suggested that public health efforts could be targeted towards specific regions and populations that experience higher HPV prevalence. Furthermore, by conducting individual proportional meta-analyses based on anatomical regions, the authors concluded that HPV (in terms of health policy) should not be approached as a problem restricted to cervical cancer [20].

This exemplar proportional meta-analysis (Fig. 1) includes all the familiar hallmarks expected when conducting and reporting a pairwise meta-analysis and serves as a benchmark for the characteristics required when presenting the results of a proportional meta-analysis. The analysis clearly presents the individual study estimates, the individual weight that each study is contributing to the pooled estimate, the pooled estimates with associated 95% confidence intervals, and the statistical tests for heterogeneity. The authors have also provided the pooled estimates using both the fixed and random effects model.

#### Review Example 2

**Fig. 2:**
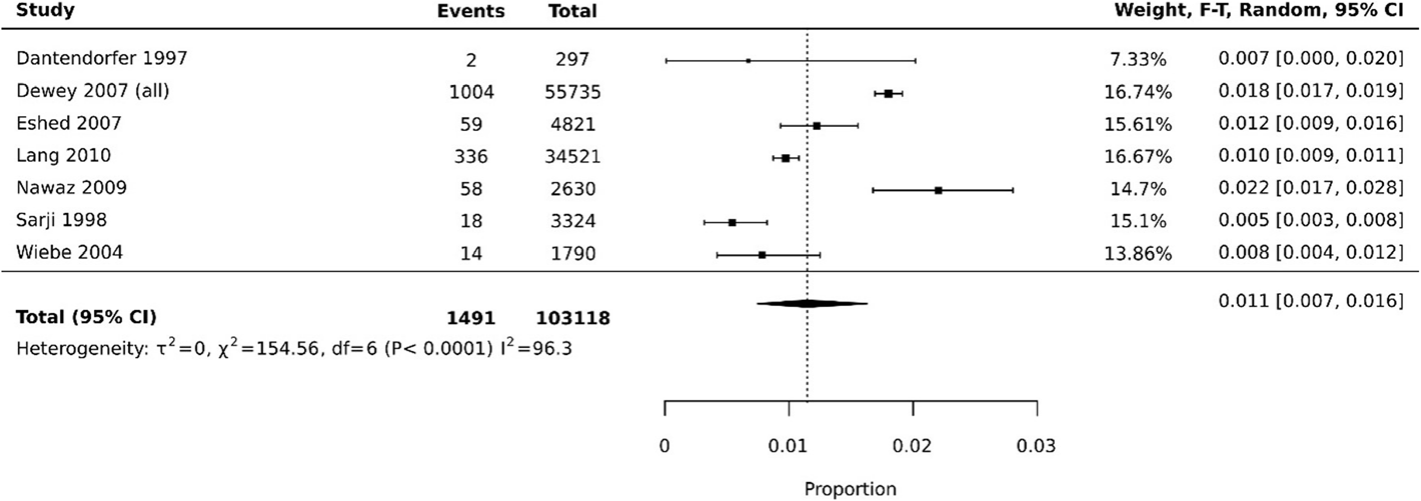
Example prevalence proportional meta-analysis of events, assessing MRI scan terminations or refusals due to claustrophobia. Recreated in JBI SUMARI software using data from: Munn, Z et al. “Claustrophobia in magnetic resonance imaging: a systematic review and meta-analysis”. Radiography 21.2 (2015): e59-e63 [12]

Another example of a forest-plot presenting a proportional meta-analysis can be seen in Fig. 2. This forest-plot has been recreated in JBI SUMARI [21] using data from a systematic review investigating premature scan termination or refusal due to claustrophobia in magnetic resonance imaging (MRI) [12]. This review included 18 studies (7 studies included in synthesis) and data from more than 100,000 MRI scans.

Each black square represents the proportion of premature scan terminations reported in each study. The pooled prevalence is 0.011, or 1.1% (95% CI 0.007–0.016), indicating that of all MRI scans conducted in the synthesised studies, 1.1% of them were terminated prematurely due to claustrophobia. The authors summarised this data by stating that health professionals working in MRI departments need to be prepared to encounter claustrophobic patients and that alternative MRI scanners (wide bore MRI scanners or open MRI systems) may be an appropriate treatment option for claustrophobic patients. The data synthesised using this proportional meta-analysis may be useful for MRI departments, funders, and clinicians to inform the efficient planning of services.

### Overall prognosis

Proportional meta-analysis can be a useful tool for providing estimates of overall prognosis, such as the expected mortality from a particular disease. These questions are very similar to prevalence/incidence questions, and many of the methods and ideas underpinning overall prognosis crossover with those of prevalence and incidence systematic reviews. For example, the Centre for Evidence-based Medicine have produced a proportional meta-analysis on mortality from COVID-19 [22].

### Systematic reviews of interventions and therapies

Although not common, proportional meta-analyses can also be used in systematic reviews addressing the effectiveness of treatment or interventions. Whilst unable to provide causal information regarding the effectiveness of a treatment, in fields where rigorous comparative studies are lacking, proportional meta-analysis may be useful to summarise the impact of a treatment on a particular condition [23]. This type of information may come from pre-test post-test studies, case series, or a single arm of a randomised controlled trial or other comparative study. Although this type of estimate will likely provide lower certainty than if coming from a comparative meta-analysis, it may still be useful to inform decision making particularly in the absence of higher quality evidence, especially in fields such as surgery where randomised controlled trials are rare [24] (amongst others) (Review Example 3). This principle is true also for pre-clinical studies where investigations may have been performed on a single group in the treatment discovery and testing phase [25, 26].

#### Review Example 3

**Fig. 3:**
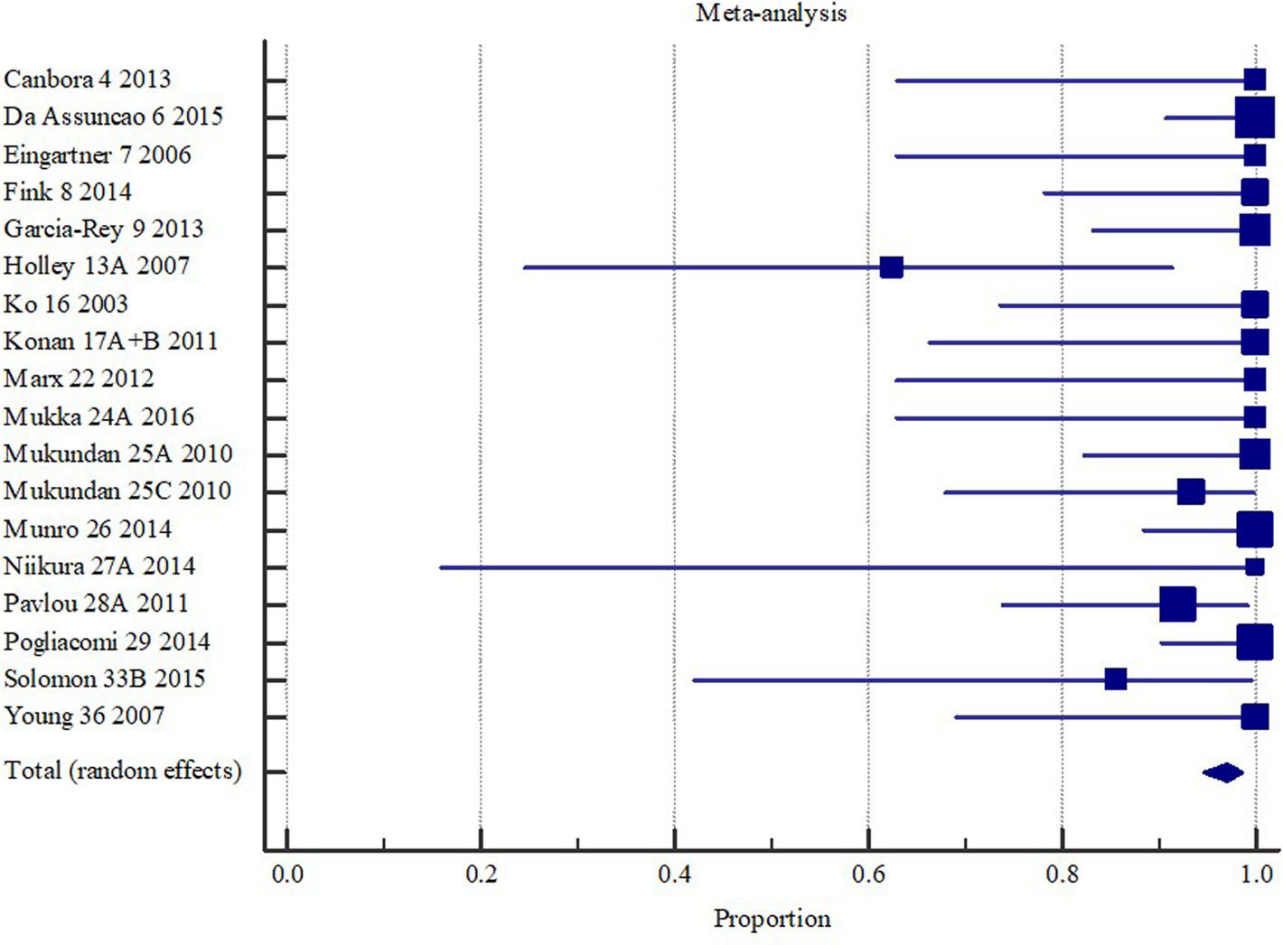
Union (overall) with revision surgery with or without wires/ cerclage/ cables following hemi or total hip arthroplasty. Created in MedCalc software from: Ianunzio, I et al. “Effectiveness of operative interventions in individuals with a hemi or total hip arthroplasty who sustain a Vancouver B2 peri-prosthetic femoral fracture” [Thesis] The University of Adelaide (2018) [27]

In an unpublished systematic review assessing the effectiveness of operative interventions in individuals with a hemi or total hip arthroplasty who had sustained a peri-prosthetic fracture, revision surgery was found to achieve union in 97% of the sampled patients (Fig. 3) [27].

**Fig. 4:**
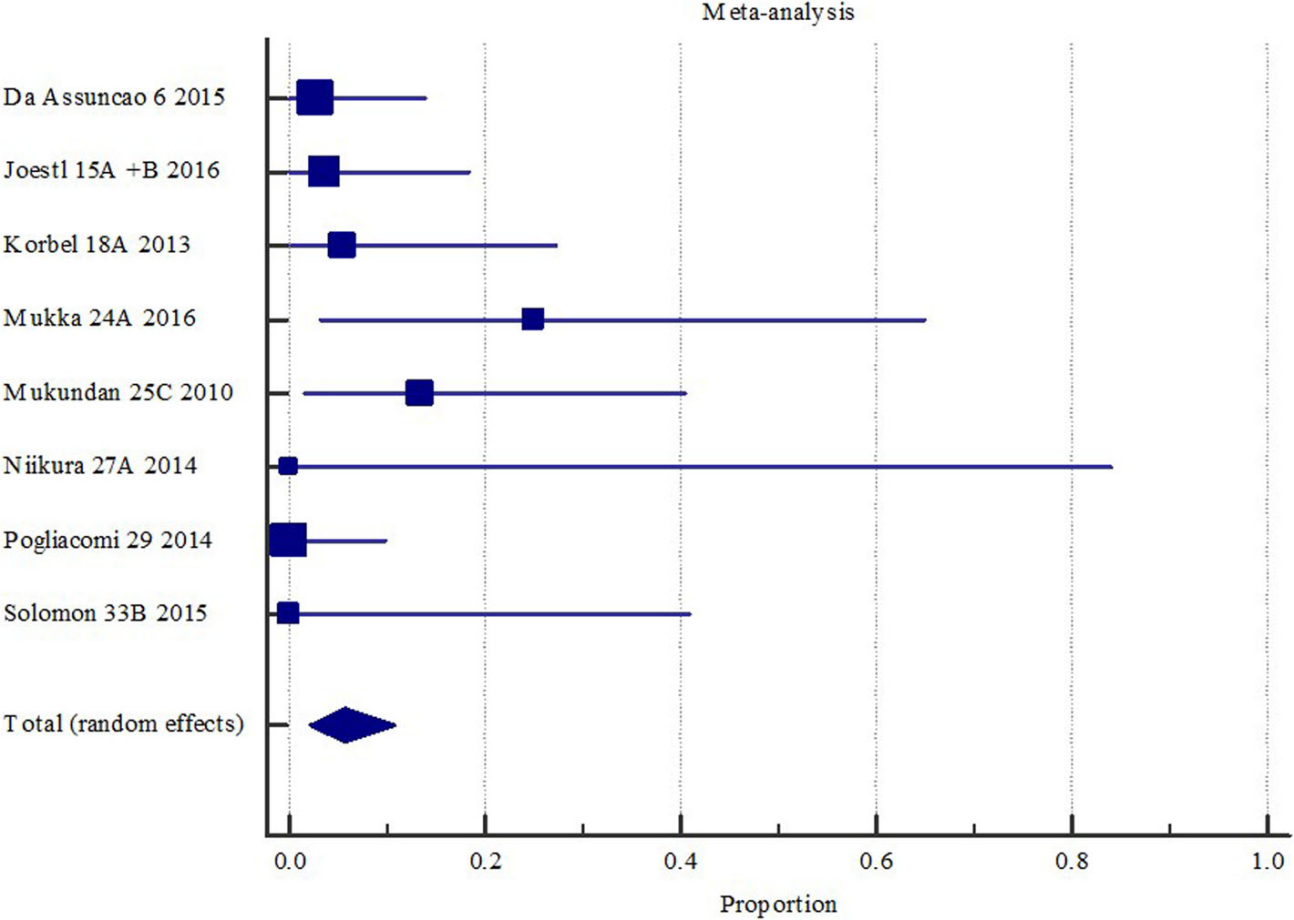
Deep surgical site infection with revision surgery with or without wires/ cerclage/ cables following hemi or total hip arthroplasty. Created in MedCalc software from: Ianunzio, I et al. “Effectiveness of operative interventions in individuals with a hemi or total hip arthroplasty who sustain a Vancouver B2 peri-prosthetic femoral fracture” [Thesis] The University of Adelaide (2018) [27]

Benefits from these types of analyses include support of decision aids and shared decision making to enable the patient to know what they can expect following surgery [28]. For example, the same review as discussed above identified a 5.7% chance of deep surgical site infection following revision hip replacement surgery, as shown in Fig. 4 [27].

### Diagnostic test accuracy reviews

Although not common and strongly advised against, proportional meta-analysis has been applied in certain diagnostic test accuracy reviews for producing summary estimates of sensitivity and specificity, in a coupled proportional meta-analysis. This has historically been justified as appropriate when the same diagnostic threshold is used across all studies included in the analysis and there is little statistical heterogeneity observed [22, 29, 30]. When diagnostic thresholds vary, diagnostic test accuracy across studies should be analysed using a summary receiver operating characteristic curve. However, this separate pooling of sensitivity and specificity via proportional meta-analysis does not account for the relationship between specificity and sensitivity, which can lead to inaccurate estimates of test accuracy [31]. Bivariate models or hierarchical approaches are necessary for this type of analysis [32–34]. As such, despite proportional meta-analysis being technically possible in this scenario, we advise systematic reviewers conducting reviews of diagnostic test accuracy to avoid proportional meta-analysis. When reading a review of diagnostic test accuracy where pooled estimates have been provided in a coupled meta-analysis of sensitivity and specificity, the results should be interpreted with caution.

### Models and formula

Incidence and prevalence are reported in terms of their proportions (discussed above). As described by Barendregt, Doi [4] this implies two important statistical considerations. The first, is that prevalence/incidence will always fall between the values of zero and one. The second, is that the sum of the prevalence/incidence over different categories should always equal one [4]. These rules are important when considering the pooling of proportional data to include in a proportional meta-analysis. Without transformation of the included proportional data the accompanying meta-analyses experience two threats to statistical conclusion validity [35–37]. Firstly, the confidence limits fall outside of the established zero to one range [4]; this may impact on the readability and presentation of the pooled data as a forest-plot. The second concern, and by far the most prudent, is that the variance from studies contributing proportional data at the extreme ends of the zero to one range tends toward zero [4]. This in turn, artificially inflates the weight that these studies contribute towards the pooled-prevalence estimate. Transformation of that data is therefore required during the meta-analysis process to deal with these problems [4, 16]. The two most common methods for performing this transformation are the double arcsine transformation (Freeman-Tukey transformation) and the logit transformation [4]. Both of these transformations calculate a pooled prevalence estimate with a 95% confidence interval under both the fixed and random-effects model. While the logit transformation solves the problem of confidence interval estimates falling outside the zero to one range, it does not necessarily resolve the issues regarding variance from extreme proportional datasets. As the double arcsine transformation addresses both problems listed above it is the preferred transformation method when performing proportional meta-analysis. Once the meta-analysis has been performed on the transformed proportions, a back-transformation is required. For log, logit, and arcsine methods, the back-transformation is straightforward. However, for the Freeman-Tukey double arcsine method there is still no consensus about which back-transformation method should be used [38]. A detailed breakdown and review of transformation and back-transformation methods can be found in the seminal prevalence meta-analysis work from Barendregt, Doi [4]. It is important to note that transformation methods may be dispensable when proportions for studies are close to 50%, for example, situations in which an effect or event is expected to be normally distributed.

As with traditional comparative methods of meta-analysis, there are different options in terms of model choice when performing a proportional meta-analysis (discussed above) [39]. When considering that epidemiological factors typically measured using proportional data are well-known to vary between population characteristics, it has been previously recommended that proportional meta-analyses are performed using the random-effects model [40]. When considering that the fixed-effect model assumes that there is one true estimate measured across studies, this is unlikely to hold true for proportional data synthesised from multiple independent studies. While performing a proportional meta-analysis using the fixed-effect model is possible, authors should be aware of the assumptions this model has on the data and the subsequent inferences that can be made from the final pooled-prevalence estimate [16].

### Subgroups and multiple categories

In some instances, proportional meta-analysis, like comparative meta-analysis, can facilitate the presentation of subgroups, or multiple categories (e.g. health status can be expressed as mild, moderate or severe). These cases provide further complications regarding the mode of transformation required [4, 16]. For a detailed statistical breakdown of these transformations we again direct you to the seminal paper on proportional meta-analysis from Barendregt, Doi [4] . Differences between subgroups can be compared and contrasted using the Chi^2^ test, and an example of how this can be facilitated in practice can be observed in the work of Righy, Rosa [41]. and is discussed in detail below.

### Heterogeneity

Since there are no specific tests to assess heterogeneity in proportional meta-analysis, we currently suggest the judicious use of the I^2^ statistic, noting that there are some issues with its application with these types of data. This statistic describes the variability between proportions between each subgroup expressed as a percentage [42]. Although I^2^ was developed in the context of comparative data, it is commonly applied to estimate heterogeneity for proportional meta-analysis [43, 44]. In this type of analysis, I^2^ is usually high. This can be due to the nature of proportional data, where little variance is observed even in studies with small sample size. Moreover, true heterogeneity is expected in prevalence and incidence estimates due to differences in the time and place where included studies were conducted. Therefore, high I^2^ in the context of proportional meta-analysis does not necessarily mean that data is inconsistent. As such, the results of this test should be interpreted conservatively. The chi squared test and Tau squared can also be used to investigate heterogeneity.

#### Prediction intervals vs confidence intervals

In a proportional meta-analysis, confidence intervals represent the expected average estimate of all possible studies. There are different methods to estimate the confidence interval for binomial proportions, such as Wald, Wilson-Score, and Clopper Pearson [45]. Whilst the Wald method is frequently used, it may not always be appropriate, particularly in cases with small numbers of patients or extreme estimates. In these cases the Wilson-Score and Clopper Pearson are better options [45]. Further statistical guidance and discussion about these methods can be found in the foundational work by Newcombe [46] and Brown, Cai [47].

The prediction interval estimates the true treatment effects that can be expected in future analysis, considering different settings [48]. As discussed before, we expect different point estimates for proportional data, especially for incidence and prevalence. With high heterogeneity, prediction intervals will be wider than confidence intervals, and can be considered a more conservative way to incorporate uncertainty in the analysis. Where possible, we suggest the estimation of prediction intervals alongside with confidence intervals, especially for prevalence and incidence estimates.

### Publication Bias and funnel plots

Tests to evaluate publication bias, such as Egger’s test [49], Begg’s test [50] and funnel plots, were developed in the context of comparative data. They assume studies with positive results are more frequently published than studies with negative results.

Even though it is possible to conduct these tests for proportional meta-analysis, there is no evidence that proportional data adequately adjusts for these tests. Moreover, the assumption of positive results being more often published is not necessarily true for proportional studies, since there is no clear definition or consensus about what a positive result in a meta-analysis of proportion is. Therefore, we do not recommend these analyses for proportional meta-analyses and advise that publication bias be assessed qualitatively.

### Software and packages

There are a number of software packages available to support systematic reviewers aiming to conduct a proportional meta-analysis, including JBI SUMARI [21], MedCalc, StatsDirect, MetaXL [4], STATA and R packages. In terms of selecting a particular program, systematic review authors should consider the learning curve associated with some packages, formulas and methods available in the various packages and financial constraints.

### Interpreting a proportional meta-analysis forest plot

**Fig. 5:**
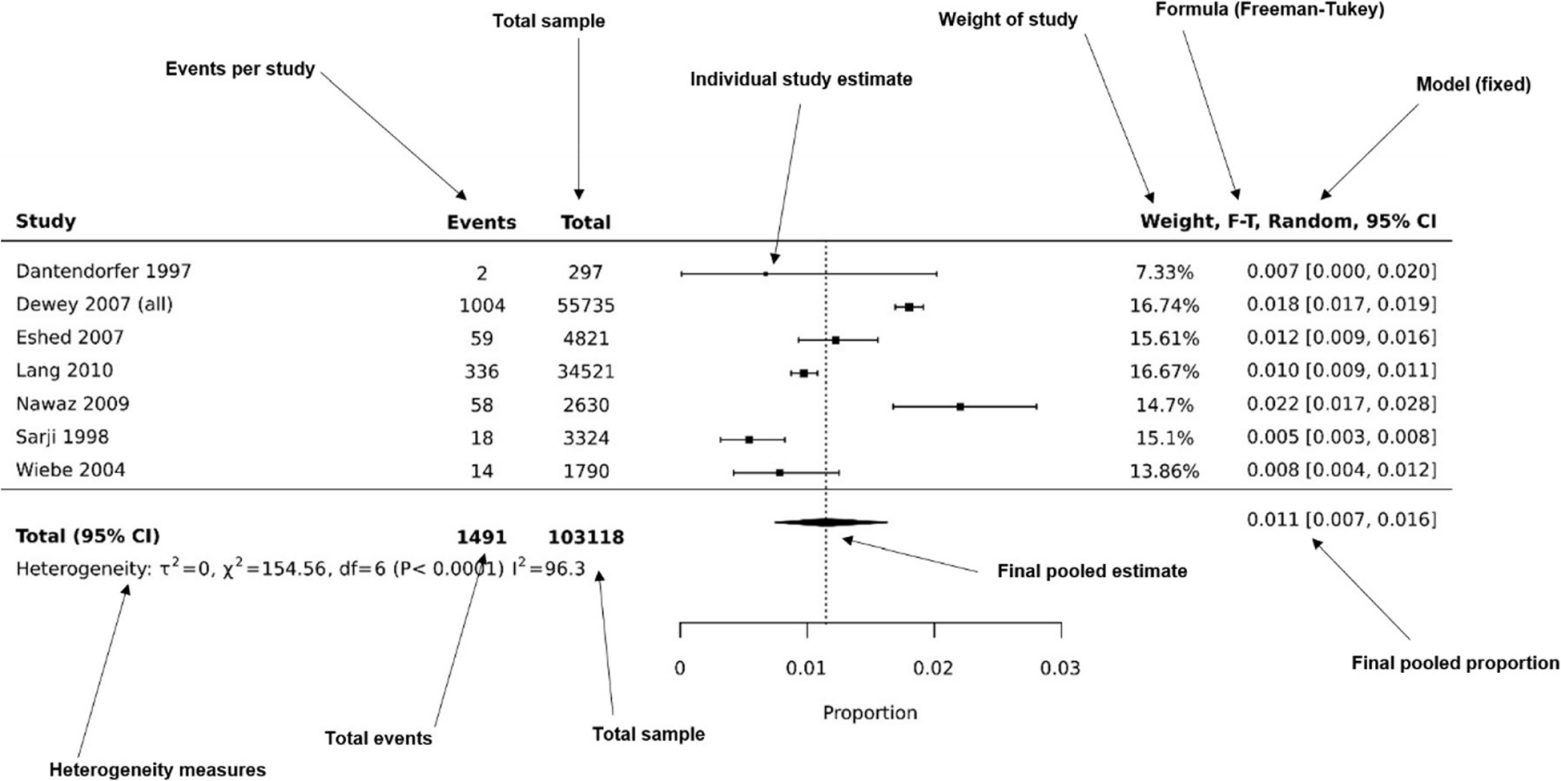
Example prevalence proportional meta-analysis of events, assessing MRI scan terminations or refusals due to claustrophobia. Annotated labels highlight key features that should be presented in a proportional meta-analysis and illustrative forest plot. Figure recreated in JBI SUMARI software using data from: Munn, Z et al. “Claustrophobia in magnetic resonance imaging: a systematic review and meta-analysis”. Radiography 21.2 (2015): e59-e63 [12]

Depending on the software or package used, each proportional meta-analysis forest plot may appear somewhat differently. However, all good packages should provide the summary estimates from each study (and their confidence interval), the final pooled proportional estimate and the associated confidence interval, measures of heterogeneity, the formula and model used, the weight of each study and ideally, the actual numbers of events and total group sizes. In Fig. 5 below, we highlight these key features in an example meta-analysis produced using JBI SUMARI. To assist in interpretation of the pooled estimate of proportions, it is perhaps simplest to communicate these results as a percentage, such as in the example below where the pooled prevalence is 0.011 (95% CI 0.007–0.016), indicating that 1.1% of population experience the event of interest.

## Conclusion

Proportional meta-analysis is a useful method for pooling data in evidence synthesis. This method is encouraged in the conduct of systematic reviews of prevalence and incidence and also interventions and therapies where appropriate. This paper has provided a high-level overview as to the methods and requirements for conducting and presenting proportional meta-analyses. When performed and interpreted correctly, proportional meta-analyses can provide systematic reviewers and evidence synthesisers answers to questions that have previously been under-investigated.

## Data Availability

All data generated or analysed during this study are included in this published article.

## Abbreviations

HPV: Human Papillomavirus
MRI: Magnetic Resonance Imaging
CI: Confidence Interval
